# The Contribution of Paresis, Age, and the Effect of Short Training on Cognitive–Motor Dual-Task Interference After Stroke: A Pilot Study

**DOI:** 10.3390/life15121881

**Published:** 2025-12-09

**Authors:** Judit Málly, Orsolya Karácsony, Bernadette Kálmán, Trevor W. Stone

**Affiliations:** 1 Institute of Neurorehabilitation, 3, Major Ln, H-9400 Sopron, Hungary; 2 Hungarian University of Sports Science, 42-48 Alkotás St., H-1123 Budapest, Hungary; 3 Laboratory of Molecular Pharmacology, HUN-REN Institute of Experimental Medicine, 43, Szigony St., H-1083 Budapest, Hungary; karacsony.orsolya@koki.hu; 4 Research Documentation Centre, HUN-REN Centre for Social Sciences, 4, Tóth Kálmán St., H-1097 Budapest, Hungary; 5 Office of the Dean and Szentagothai Research Center, School of Medicine, University of Pécs, 20, Ifjúság St., H-7624 Pécs, Hungary; 6 Kennedy Institute of Rheumatology, University of Oxford, Roosevelt DR., Oxford OX3 7FY, UK

**Keywords:** cognitive–motor dual-task test, stroke, rehabilitation, Dividat Senso, cognitive ability

## Abstract

Simultaneously performing cognitive and motor tasks after a stroke interfered with each other. Considering the competing deficits of cognition and motor paresis, we aimed here to assess the overall functional impairments of patients after stroke injury. A range of dual-task (DT) assessments was made on 63 post-stroke patients (PS) and 49 healthy age-matched controls. Patients with paresis (P) and without paresis (NP) were compared with controls before and after DT training. Differences between the NP patients and controls confirmed the cognitive decline, while the comparison between the NP and P patients strengthened the motor damage in P patients. The elderly patients performed worse. According to the ArtANOVA analysis, age was more important than paresis in DT performance. Short-term training modified the test results, especially in P patients. In conclusion, paresis and older age significantly worsen the outcomes of the cognitive dual-task tests. The age-dependent results may reflect cognitive decline, especially in NP patients. Consequently, the dual-task test results may represent global cognitive deterioration after stroke. Short-term dual-task training improves dual-task performance, especially in the P groups.

## 1. Introduction

Using dual-task (DT) activity testing, the simultaneously performed cognitive and motor tasks interfere with each other and provide a physiologically relevant indication of overall function [1,2,3]. The interference of the two tasks influences completion time and/or the number of mistakes in the cognitive task [4,5,6,7]. In most of the previous studies, walking was used as a motor task, for example, coupled with counting backwards [8,9,10,11]. In these studies, generally, gait speed, step length, and the values of dual-task completion times were analyzed. Dual-task performance has been extensively studied in the elderly [12,13,14,15,16,17], in patients with Parkinson’s disease [18,19], balance disorders [14,20], and multiple sclerosis [21,22], and in children with cerebral palsy [23].

In DT performance, attention and decision-making are sensitive indicators of cognitive decline, confounding outcomes in elderly persons [1,12,13]. Thus, for example, a reduced DT performance in patients with Parkinson’s disease (PD) may be due to cognitive decline, not bradykinesia [6,7]. Other authors have reached the same conclusion regarding decreased CM dual-task activity when testing repetitive elbow [24] and noting altered gait characteristics during walking [13] may indicate cognitive insufficiency rather than motor impairment. However, the previous assessment of DT performance could not unequivocally distinguish between motor and cognitive abilities.

The PS state is generally characterized by motor impairment, balance instability, and cognitive deterioration [25,26]. Publications with CM dual-task performances have observed an increased interference in PS patients [27,28,29,30]. The interaction of the motor and cognitive tasks was indirectly justified by the load of motor and cognitive activity. The increased motor load with walking backwards or crossing obstacles led to an increased number of cognitive errors [31]. Similarly, the loaded cognitive function resulted in slower walking, with more steps taken in the Time Up and Go Test while counting backwards [10]. The Norwegian Cognitive Impairment After Stroke analysis found a regression line between motor and cognitive impairment [32]. It is unknown whether cognitive impairment after stroke can be measured at all by DT activity. Our goal was to study patients with and without motor impairment, i.e., patients after stroke, in order to address this limitation. Most previous studies included PS patients with mild gait impairment. In our study, the range of the disabilities of PS patients was expanded in order to demonstrate the effect of mobility limitation and age on CM dual-task performance. Changes in mobility of post-stroke (PS) patients were compared between P and NP patients and between patients and normal subjects.

Several previous studies applied effective, long-term training programs, whereas short-term training is neglected [16,17,33,34,35]. Our goal was to find a time-saving, effective, easy-to-repeat activity that can influence both cognitive and motor function in PS. Therefore, we studied the effects of STDTT.

## 2. Materials and Methods

### 2.1. Study Design

Trial registration can be found at https://www.isrctn.com/ISRCTN12362945 (accessed on 14 February 2023).

**Figure 1:**
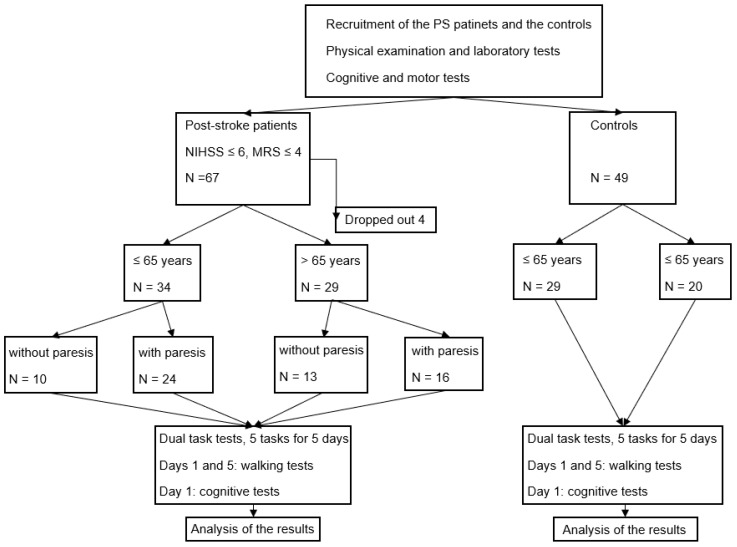
Flowchart. The diagram shows the interventions and the changes in the number of participants.

This was a comparative, prospective study. The cognitive–motor DT (CMDT) performance was measured in PS patients and controls to separate the effects of limited movement and age-dependent cognitive decline. The effect of an STDTT was determined (Figure 1).

### 2.2. Participants

The study was not randomized. Patients were recruited sequentially from within Hungary. On arrival, they were assigned to the paretic (P) or non-paretic group (NP) according to their motor assessments during the past 3 years. Sixty-seven PS patients were recruited between 1 January 2019 and 31 December 2023. Four of them fell out, leaving sixty-three patients with PS (F/M: 20/43) (Figure 1). Forty-nine healthy age-matched controls (F/M: 43/6) were recruited from the patients’ and staff relatives.

Inclusion criteria for PS patients included one stroke event, no evidence of dementia (normal Mini-Mental State Examination), and the ability to stand and walk unaided. Patients with receptive aphasia were excluded. A National Institute of Health Stroke Scale (NIHSS) score of 6 or less and a modified Rankin Scale (mRS) score of 4 or less were required.

Subjects had suffered mild to severe levels of stroke damage, including 51 who had a brain infarct caused by arterial occlusion, 12 who had a hemorrhage in the basal ganglia, and 1 person who had a cerebellar hemorrhage. Comorbidities included 45 cases of hypertension, 18 cases of diabetes mellitus, and 7 cases of atrial fibrillation. PS patients were also divided into P and NP groups, with the latter having mild symptoms, like sensory disturbance or mild ataxia.

### 2.3. Assessments

Dual-task performances were assessed using Dividat Senso equipment (HUR, Finland) [7], an online program regularly checked, serviced, and updated by Joris van het Reve Dividat AG, Switzerland, and all data were recorded automatically by the computer.

Subjects stood on a glass platform (106 cm × 106 cm) overlying 20 force sensors to measure both sole pressure and body movement. Three sides of the platform were surrounded by a railing to support participants. Subjects viewed a test game on a monitor and reacted by leg movements when they perceived any edge of the screen, where five DT tests were applied, and they had to react with four-way leg movements to the objects appearing on the screen. The tests needed attention and decision-making and included a visual and an abstraction task.

The ‘Simple’ task involved selecting a red dot, while the ‘Bird’ test required choosing a ‘Bird’ from differently colored figures. A red dot and different melodies were combined in the ‘Divided’ task, and the ‘Habitat’ task required assigning different animals to their habitat. In the ‘Target’ task, black balls moved at different speeds, and when they reached the target, a step was required from the participant. The number of correct ‘Hits’ and incorrect ‘Misses’ was recorded. The tasks lasted for one and a half minutes and were repeated on five consecutive days.

The following traditional tests were also applied: Mini-Mental State Examination (MMSE) [36], the Ziehen–Ranschburg Word Pairs Test, the Trail Making Test [37], the Clock Drawing Test [38], and the Hamilton Depression Scale Tests [39], and for the quantitation of post-stroke disability, the NIHSS [40] and mRS were used [41]. Upper and lower extremity strength was rated on a 5-point scale from 0 (no movement) to 5 (normal strength). Total points were calculated as a sum of points from every movement of the paretic limbs. Walking ability was measured on days 1 and 5 of training at a distance (m) for 6 min, and the time (s) to walk 10 m on a designated (25 m), covered, smooth, and supervised surface was also measured. Test supervisors were blinded to the grouping of the participants. The tests took a total of one hour. The daily gymnastics performed at home by participants were not supplemented with additional physiotherapy. Other activities were excluded during the training with dual-task performance.

### 2.4. Statistical Analysis

The modified Rankin Scale (mRS) is an ordinal clinical scale. In line with this, mRS values in this study were used only for descriptive clinical characterization of the stroke cohort, and no parametric statistics or inferential tests were applied to this measure.

The results are expressed as the mean ± standard deviation. The normality of data from the Bird test was determined by the Shapiro–Wilk test in OriginPro 2023b (OriginLab Corporation, Northampton, MA, USA). Data from 9 of 12 groups (based on age, presence of paresis, and day of examination) were not normally distributed [42], so the non-parametric Mann–Whitney U test was used to assess differences in data in patients and control subgroups. Effects of age and paresis on days 1 and 5 of the experiment were calculated using the Analysis of Variance of Aligned Rank Transformed Data (ArtANOVA) in R v.4.3.2. For post hoc comparisons of the main effects, we used the partial η^2^ effect size statistics, interpretating their values as a small effect for 0.01–<0.06, as a medium effect for 0.06–<0.14, and as a large effect for ≥0.14, following the recommendations of Cohen [43]. The number of the minimal necessary cohort size was determined to be 43 by statistical power analysis using G*Power in OriginPro 2023b (10.05, Northampton, MA, USA). The number of actual participants was 63.

## 3. Results

### 3.1. Characteristics of PS Participants

#### 3.1.1. Cognitive Tests

According to the MMSE test, no participant showed signs of dementia or severe working memory disturbances.

#### 3.1.2. Walking Tests

None of the six-minute walking test nor the time needed for the 10 m walk showed significant changes comparing the initial and 5-day post-training results (Table 1(A,B)).

#### 3.1.3. Stroke Assessment

A National Institutes of Health Stroke Scale (NIHSS) score and a modified Rankin Scale (mRS) score were recorded for descriptive purposes. The observed differences between subgroups reflect clinically relevant variability but were not used in inferential analyses.

NIHSS values were significantly different between the two age groups of NP patients (*p* = 0.047) but not of P patients (*p* = 0.086). Neither the upper (*p* = 0.30346) nor the lower extremity scores (*p* = 0.256448) differed between the two age groups (Table 1(A,B)).

### 3.2. Comparisons of the DT Results Between the P or NP PS Patients and Controls

**Figure 2:**
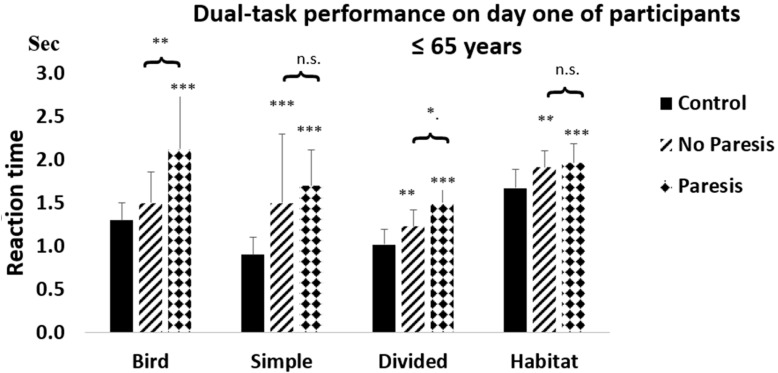
Dual-task performance on day one of participants ≤ 65 years. The figure demonstrates the differences between controls and NP and P patients with post-stroke (PS) ≤ 65 years on the first day of the study. The asterisks (*) indicate significant differences between the controls and the PS patients ≤ 65 years. ** indicates the level of significance (*p* < 0.01) between the control group and the NP PS patients. *** shows the significance (*p* < 0.001) between the control group and the P group of patients. The clips show the difference between the NP and P groups. The * means (*p* < 0.05) in the dual-task test (Dividat) and ** shows the level of significance (*p* < 0.01) in the test of Bird. With the exception of ‘Bird’, a remarkable delay in reaction time was observed in both the NP and the P groups. Columns represent the mean ± SD. The columns were labeled as follows: control with black, NP group with oblique striped lines, and P group with dotted patterns. The figure was created by Microsoft Excel.

**Figure 3:**
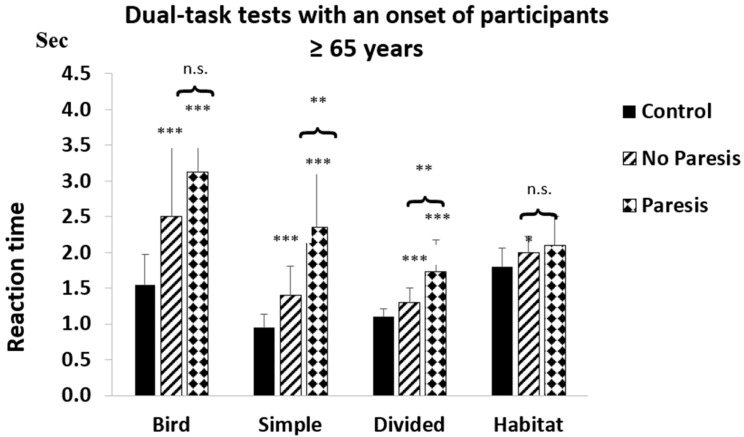
Dual-task tests of the participants with an age of ≥ 65 years. The figure depicts the differences between the controls and NP and P patients with post-stroke (PS) > 65 years on the first day of the study. The asterisks (*) indicate significant differences when the controls are compared with PS patients > 65 years. *** shows the level of significance (*p* < 0.001), * = *p* < 0.05. The clips show the difference between the NP and P groups. In two dual-task tests (Simple and Divided) the difference was ** = *p* < 0.01. A remarkable delay in the reaction time was observed in both the NP and the P groups. There was no interference in ‘Habitat’. Columns represent the mean ± SD. The columns were labeled as follows: control with black, NP group with oblique striped lines, and P group with dotted patterns. The figure was created by Microsoft Excel.

Compared with controls, the reaction times of the NP or P groups were significantly increased, showing significant increases for patients at or below 65 years, except for controls vs. NP comparisons in the Bird test. Reaction times were significantly increased in the NP and P vs. control comparisons in all tests, except in the Habitat test, in the >65 years age group (Figure 2 and Figure 3).

The decline in DT performance is primarily defined by the deterioration of cognitive function in the NP group, where there is no limitation of movement. On the first day of testing, reaction times in the NP and control groups below 65 years of age were significantly different for three tests: Simple (*p* < 0.001), Divided (*p* < 0.01), and Habitat (*p* < 0.01) (Figure 2).

Reaction times in the NP and control groups above 65 years of age were significantly different in all tests. For Habitat (*p* < 0.05 (in Figure 3)), this is a non-significant trend, and for other tests, it is significant (*p* < 0.001 (Figure 3)).

Comparing the number of Hits, a significant decrease was noted in the NP and P groups vs. controls in the over-65 group controls (*p* < 0.001), and an increase in Misses was detected in the NP (trend) or P (*p* < 0.001) (Table 2 and Table 3).

Table 2 shows the results of the dual-task tests of PS patients and the control group on day 1 compared with the results on day 5 after dual-task training. In the control group, and especially in the paretic (P) PS group under 65 years, the values changed significantly on day 5 compared to the data of day one. The non-paretic (NP) PS group showed alteration in two dual-task tests. The results of the ‘Misses’ did not change significantly, except for the P group; however, the decreased value did not reach the control results.

Table 3 reflects the results of the dual-task performances of post-stroke patients (PS) and the control group on day 1 compared with the results on day 5 after dual-task training. The results of controls and non-paretic patients (NP) changed into three different dual-task performances by the end of the fifth day. Changes after dual-task training were observed to be highly significant in the paretic group of PS patients.

#### 3.2.1. Comparisons of the DT Results in the NP and P Patients’ Groups

The effect of paresis on DT performance was estimated by comparing the P and NP groups. At the beginning, the difference was significant for three tests in both age groups. Below the age of 65, this was the Bird (*p* < 0.01) and the Divided (*p* < 0.05) (Figure 2). Over the age of 65, the Simple (*p* < 0.01) and the Divided tests were different (*p* < 0.01) (Figure 3).

Although there was also a trend of an increase in Hits and a decrease in Misses when P and NP patients were compared on day 5 for the over-65 groups, this was not significantly different (Table 3).

#### 3.2.2. Comparison of Participants’ DT Test Results on Days One and Five

Significant differences were seen in the DT performances of P and NP PS patients when the 5-day post-training and the first-day results were compared. While there was a significant post-training improvement in the ‘Simple’ and ‘Habitat’ tests in NP patients under 65 years, the post-training results in the P group improved in the ‘Bird’, ‘Simple’, ‘Hits’, and ‘Misses’ tests (Table 2).

In P and NP patients over 65 years of age, the P patients improved significantly in more DT tests than NP PS patients after 5 days of dual-task training (Table 3). The results of NP patients above 65 significantly improved in the Simple (*p* < 0.05), Divided (*p* < 0.05), and Target (Hits: tests (all *p* < 0.05)) tests. For P patients, the results significantly improved in the Bird (*p* < 0.01), Simple (*p* < 0.001), Divided (*p* < 0.05), and Target (Hits: *p* < 0.05) tests (Table 3).

#### 3.2.3. Contribution of Age and Paresis to Performance in CMDT Tests

The CMDT activity was influenced by paresis and age, which should be considered at each examination. The contribution of paresis and age was assessed by ArtANOVA. The effect reflects the strength of the relationship between two variables, or a sample-based estimate, which is eta square. It was significant for age in the Bird (η^2^ = 0.563), Hits (η^2^ = 0.468), and Misses (η^2^ = 0.285) tests and significant in the Simple (η^2^ = 0.118) and Divided (η^2^ = 0.141) tests on the first day of study. The influence of paresis was significant in the Bird (η^2^ = 0.115), Simple (η^2^ = 0.176), and Divided (η^2^ = 0.183) tests. At the end of the training period, the effect of age on CMDT tests remained significant in Misses (η^2^ = 0.0270) and highly significant in all other tests

(Bird: η^2^ = 0.346, Simple: η^2^ = 0.435, Divided: η^2^ = 0.390, Habitat: η^2^ = 0.388, Hits: η^2^ = 0.566), while paresis did not influence the dual-task performances at this point.

The ArtANOVA analysis helped to clarify the ages at which cognition was impaired or movements became restricted, which played a role in DT performance.

## 4. Discussion

The novelty of our study was the comparison of NP and severely affected P PS patients to assess the influence of paresis, age, and training before and after training. Cognitive decline was indicated by the reduced DT performances in PS patients, especially when NP participants and controls were compared. The importance of paresis in CMDT tests was apparent in both PS age groups, confirmed by comparing P and NP PS patients using the ArtANOVA analysis.

The importance of paresis in the outcome of CMDT tests was apparent in both PS age groups and was confirmed by comparing NP and P PS patients using the ArtANOVA analysis. Elderly patients without motor limitations showed a greater decline in DT performance.

### 4.1. The Impact of Paresis

The effect of paresis does not dominate over cognitive decline in DT performance. Previous studies reported poor performance on CMDT tests in PS patients with mild motor impairment and noted the impact of paresis with the change in walking speed [11,27,28,30,44,45]. However, evaluation of walking is a complex task, influenced by balance [46] and training [17]. Walking speed in DT testing correlates with a decline in cognitive ability from mild (MCI) to severe cognitive impairment [9,13]. Consequently, paresis cannot be measured directly by walking; it is only an indirect indicator of paresis [46]. Conversely, it is hard to define, especially in P groups, the extent to which the two factors are responsible for the deterioration of DT performance. Cognitive impairment tends to increase in parallel with the extent of motor damage [32]. In the present work, PS patients were sorted into NP and P groups (Figure 2 and Figure 3), and performance differences were confirmed by the ArtANOVA analysis on the first day of testing. There was some reduction by SDTT on CMDT, suggesting a learning process and that paresis does not play an important role in DT performance.

### 4.2. The Impact of Cognitive Function

Some studies have reported that elderly persons with MCI performed worse in DT tests than those without it [9,32,47,48,49], although training and prioritization improved DT performance [50,51]. In contrast, the decline in DT performance may indicate cognitive impairment in the early stages of PD [6,52]. Similarly, the non-walking DT test in the upper arm repetitive elbow flexion was useful to detect cognitive decline [24].

Reduced DT performance in NP patients allowed us to predict cognitive decline in PS patients without paresis and in elderly subjects (Figure 2 and Figure 3). DT tests assessed by Dividat represent a simple, objective test, mainly influenced by attention and decision-making. These factors deteriorate with advanced age when two tasks are performed simultaneously, since cognitive demand is increased during DT performance. These two aspects of the CMDT test cannot be separated, but the DT tests provide an overall view of cognitive function. They reveal that attention deficit increases with advanced age when two tasks are performed simultaneously [53]. Cognitive demand is increased during DT performance, leading to a worsening of the outcome. These two parts of the CMDT test cannot be separated, but the DT tests provide an invaluable overall view of cognitive function in the absence of movement impairment.

The elevated number of ‘Mistakes’, indicating cognitive decline, could not be influenced by training. According to our statistical analysis, the measure of ‘Mistakes’ was independent of paresis and highly dependent on age. An increase in ‘Mistakes’ was observed neither in the NP groups under age 65 nor in the control subjects (Table 2 and Table 3). These results are in contradiction with previous results [15], although our result confirms the observation that cognitive DT test errors increased only in PS patients but not in controls [9].

### 4.3. The Impact of Age

Age-related decline in DT performance was observed in three different age groups, which was explained by the difficulty in dividing attention between the two tasks [47]. Cognitive resources are overloaded by the dual task, reducing dual-task performance [48]. In our study, there was no difference in the age distribution of controls and the NP patients under age 65. Our results are in contradiction with the results reported by Bech et al. and Brustio et al. [15,47]. These authors observed a decrement in healthy elderly persons, whereas our study only demonstrated a reduction in DT performance in PS patients with paresis and/or the very elderly. Our observation, therefore, may suggest some degree of cognitive decline.

### 4.4. The Impact of Training

A favorable effect of DT training has been observed on walking speed, stride length, balance, and cognitive function in normal elderly people [5,17,54,55] and in PS patients [33,34,35,56,57]. Here, DT trainings were applied twice a week, for several weeks. Our data suggest that shorter training periods on consecutive days can be effective, as they reduce the delay of CMDT performances. The effect of training was particularly pronounced in the patients with paresis, although the walking test did not show an improvement after short-duration training.

Our data suggest that even shorter training on consecutive days can be effective, as it reduces the delay of CMDT performances. In this study, the effect of training was particularly pronounced in the patients with paresis, although the walking test did not show an improvement after short-duration training.

### 4.5. Limitations of the Study

This study did not use classical randomization; instead, patients were divided into different groups according to their symptoms. Due to the long-term recruitment and the strict inclusion criteria, the validity of the data was less affected. Blinding was only possible for psychological and exergaming tests. A short-duration training could have beneficially biased DT performance.

Furthermore, as the modified Rankin Scale is an ordinal measure, it was used solely for descriptive clinical characterization and was not included in any inferential statistical procedures.

## 5. Conclusions

Motor impairment significantly contributed to the decrease in DT performances in PS patients over and below 65 years. Based on the significant differences between the DT performances of the NP and control groups, some cognitive deterioration in PS patients can also be inferred, with poorer performance in elderly subjects. These results suggest the presence of attention and decision-making deficits in PS patients. Training produced improvements in the PS patients, with the greatest effect in the P group.

## Figures and Tables

**Table 1:** Summary of the demographic data and characteristics of stroke classification, cognitive status, and walking test results below and over 65 years of age. Values are presented descriptively; no parametric statistics were applied.

**(A)**			
**Demographic Data of Participants**			
**≤65 Years**			
Items	Control	No paresis	Paresis
Number	29	10	24
Age (yrs.)	55.0 ± 5.79	59.1 ± 1.12	52.3 ± 11.3
Female; male	26; 3	3; 7	6; 18
Duration of disease (yrs.)		0.57 ± 0.78	3.3 ± 1.34
NIHSS		0.57 ± 0.78	3.4 ± 1.50
Modified Rankin Scale		0.88 ± 0.92	2.56 ± 0.66
Mini-Mental Rating Scale	29.8 ± 0.56	28.3 ± 4.04	29.86 ± 0.34
Ziehen–Ranscburg Word Pairs Test	80.6 ± 13.71	64.6 ± 21.52 *	67.4 ± 22.19
Hamilton Depression Scale	5.2 ± 2.85	4.8 ± 2.04	7.5 ± 4.36
Trail Making B-A	25.9 ± 9.75	37.2 ± 31.11	24.2 ± 14.28
6 min walk	614.0 ± 125.8	453.5 ± 86.9	286.8 ± 164.3
Time needed to walk 10 m	5.8 ± 0.94	7.7 ± 2.42	21.0 ± 18.06
6 min walk, day 5		472.1 ± 67.19	286.5 ± 164.35
Time needed to walk 10 m, day 5		7.57 ± 1.90	20.75 ± 18.82
Upper limb paresis			22.75 ± 13.52
Lower limb paresis			28.83 ± 4.65
(**B**)			
**Demographic Data of Participants**			
**≤65 Years**			
Number	20	13	16
Age (yrs.)	71.4 ± 4.48	66.5 ± 11.22	69.9 ± 3.53
Female; male	18; 2	5; 8	6; 10
Duration of disease (yrs.)		1.66 ± 1.88	2.33 ± 2.97
NIHSS		1.4 ± 0.89 *	4.5 ± 1.44
Modified Rankin Scale		1.8 ± 1.30 *	3.2 ± 0.77 *
Mini-Mental Rating Scale	29.4 ± 0.89	27.8 ± 2.13	29.1 ± 1.60
Ziehen–Ranschburg Word Pairs Test	69.5 ± 14.66	51.3 ± 24.71	61.0 ± 18.9
Hamilton Depression Scale	6.31 ± 2.41	6.3 ± 3.61	9.3 ± 5.22
Trail Making B-A	43.1 ± 24.3	80.2 ± 77.95	100.8 ± 89.54
6 min walk	533.4 ± 117.34	343.8 ± 109.02	126.2 ± 55.74
Time needed to walk 10 m	6.6 ± 1.66	9.5 ± 3.28	39.4 ± 16.27
6 min walk, day 5		318.7 ± 91.71	142.3 ± 63.33
Time needed to walk 10 m, day 5		11.9 ± 5.10	39.4 ± 16.27
Upper limb paresis			19.6 ± 15.86
Lower limb paresis			25.6 ± 9.09

In the non-paretic group, NIHSS and mRS showed a significant difference between the two age groups (*p* = 0.047). There was no significant difference in the extent of upper and lower limb paresis. The results of the 6 min walk test and the 10 m walk test showed no significant change by the end of the training period. The short-memory test, the Ziehen-Ranschburg Word Pairs Test showed a significant difference between the control group and the NP participants under 65 years of age (* = *p* < 0.05).

**Table 2:** Comparisons of the dual-task test results in PS patients and controls at or under the age of 65 years on days 1 and 5 post-training.

	After Dual-Task Training Comparison of Dual-Task Tests on Days 1 and 5					
				≤65 Years		
		Day One			Day Five	
Tests	Control	Non-Paretic	Paretic	Control	Non-Paretic	Paretic
Bird	1.3 ± 0.2	1.6 ± 0.4	2.1 ± 0.7	1.0 ± 0.1 ***	1.3 ± 0.3	1.5 ± 0.4 **
Simple	0.9 ± 0.2	1.6 ± 0.8	1.7 ± 0.4	0.8 ± 0.1 ***	1.0 ± 0.2 *	1.1 ± 0.2 ***
Divided	1.0 ± 0.2	1.2 ± 0.2	1.4 ± 0.3	0.9 ± 0.2 *	1.1 ± 0.2	1.2 ± 0.2 **
Habitat	1.7 ± 0.2	1.9 ± 0.2	2.0 ± 0.2	1.4 ± 0.1 ***	1.6 ± 0.2 *	1.7 ± 0.3 *
Hits	97.8 ± 16.8	82.4 ± 18.5	54.5 ± 25.1	118.3 ± 21.1 ***	89.8 ± 19.2	76.4 ± 25.7 *
Misses	11.6 ± 4.7	15.8 ± 7.3	25.5 ± 17.6	10.3 ± 7.0	14.5 ± 7.4	17.0 ± 0.3 *

Values represent the mean ± SD. The asterisks (*) indicate significant differences between the controls, NP, and P PS patients ≤ 65 years of age on the first day and all of them on the fifth day. * *p* < 0.05, ** *p* < 0.01, and *** *p* < 0.001.

**Table 3:** Comparison of the dual-task test results in PS patients and controls beyond the age of 65 years on days 1 and 5 post-training.

After Dual-Task Training Comparison of Dual-Task Tests on Days 1 and 5						
				>65 Years		
		Day One			Day Five	
Tests	Control	Non-Paretic	Paretic	Control	Non-Paretic	Paretic
Bird	1.5 ± 0.4	2.7 ± 1.2	3.1 ± 1.0	1.2 ± 0.1 ***	1.3 ± 0.3	2.0 ± 0.8 **
Simple	0.9 ± 0.2	1.6 ± 0.4	2.3 ± 1.0	0.9 ± 0.1	1.0 ± 0.2 *	1.5 ± 0.4 ***
Divided	1.0 ± 0.1	1.5 ± 0.2	1.7 ± 0.4	0.9 ± 0.1 **	1.1 ± 0.2 *	1.4 ± 0.3 *
Habitat	1.7 ± 0.2	2.0 ± 0.2	2.0 ± 0.4	1.6 ± 0.2 *	1.6 ± 0.2	2.0 ± 0.2 *
Hits	74.3 ± 11.4	39.7 ± 17.0	28.5 ± 19.4	108.0 ± 12.7 ***	89.8 ± 19.2 *	42.9 ± 23.7 *
Misses	13.5 ± 4.2	35.7 ± 14.1	44.4 ± 19.0	11.0 ± 5.3	14.5 ± 7.4	35.0 ± 17.1

Values represent the mean ± SD. The asterisks (*) indicate significant differences between the controls, NP, and P PS patients ≥ 65 years of age on the first day and all of them on the fifth day. * *p* < 0.05, ** *p* < 0.01, and *** *p* < 0.001.

## Data Availability

Archiving of research data was supported by the Data Repository Platform of the Hungarian Research Network (HUN-REN ARP) under the ARP Ambassador program. The research data are accessed in the ARP Data Repository via this link: https://hdl.handle.net/21.15109/ARP/LZXB8R.
